# Factors Associated with Virological Non-Suppression Among People Living with HIV Receiving Antiretroviral Therapy in Kazakhstan: A National Registry-Based Study

**DOI:** 10.3390/tropicalmed11060156

**Published:** 2026-06-09

**Authors:** Anel Ibrayeva, Zhamilya Nugmanova, Anarkhan Nurkerimova, Aigerim Alimbekova, Marat Tukeyev, Alfiya Denebaeva, Jack DeHovitz, Yerlan Ismoldayev, Bolat Sadykov, Shynar Tanabayeva, Ildar Fakhradiyev

**Affiliations:** 1 Department of Medicne, S.D. Asfendiyarov Kazakh National Medical University, Almaty 050000, Kazakhstan; 2 Center for AIDS Control and Prevention, Almaty 050060, Kazakhstan; 3 Department of Medicne, SUNY Downstate Health Sciences University, New York, NY 11203, USA; 4 College of Medicine, Korea University, Seoul 02841, Republic of Korea

**Keywords:** human immunodeficiency virus, antiretroviral therapy, virological suppression, virological non-suppression, adherence, cluster of differentiation 4 cell count, Kazakhstan

## Abstract

**Background**: Virological suppression is a key outcome of antiretroviral therapy. Despite progress in HIV treatment in Kazakhstan, virological non-suppression remains a relevant clinical and public health issue requiring further analysis. This study aimed to assess the prevalence of virological suppression (VS) and to identify factors associated with the absence of VS among people living with human immunodeficiency virus (HIV) who are receiving antiretroviral therapy (ART) in Kazakhstan. **Methods**: A retrospective cross-sectional analytical study was conducted using secondary analysis of a de-identified national registry database of people living with HIV (PLHIV) receiving ART in the Republic of Kazakhstan as of 30 September 2025. The primary outcome was virological non-suppression (VNS), defined as the last viral load (VL) value of at least 200 copies per milliliter. The analysis included sex, age, presumed route of HIV transmission, the first available cluster of differentiation 4 (CD4) cell count recorded in the registry, the last recorded percentage category of adherence to ART, and the aggregated category of ART regimen. The main descriptive, bivariate, and multivariable analyses were performed using a complete-case approach. Independent associations were assessed using multivariable logistic regression, and the results were presented as adjusted odds ratios (aORs) with 95 percent confidence intervals (CIs). **Results**: The initial registry extraction included 33,614 records, of which 32,130 patients were included in the final analytical sample. VS was achieved in 29,454 (91.7%) patients, whereas VNS was observed in 2676 (8.3%) patients. In the multivariable model, higher adjusted odds of VNS were observed among men compared with women (aOR 1.14; 95% CI 1.02–1.26), as well as among patients with a first CD4 count < 200 cells/μL compared with those with a first CD4 count of ≥500 cells/μL (aOR 1.25; 95% CI 1.09–1.44). The strongest association was found for reduced adherence to therapy. Compared with adherence of at least 95%, the adjusted odds of VNS were markedly higher among patients with adherence of 85–94% (aOR 28.66; 95% CI 25.85–31.77) and among those with adherence below 85% (aOR 61.05; 95% CI 50.50–73.81). In all age groups older than 25 years, the adjusted odds of VNS were lower than among patients younger than 25 years. Lower adjusted odds of VNS were also observed among patients with homosexual transmission, vertical transmission, and other or unspecified transmission routes compared with heterosexual transmission. Among ART regimens, regimens containing non-nucleoside reverse transcriptase inhibitors (NNRTIs) were associated with lower adjusted odds of VNS than dolutegravir-containing regimens (DTG-containing regimens) (aOR 0.68; 95% CI 0.52–0.88), whereas no statistically significant differences were identified for regimens containing protease inhibitors (PIs). **Conclusions**: Despite the high overall level of VS among PLHIV receiving ART in Kazakhstan, VNS remains concentrated in clinically and programmatically important subgroups. It was most strongly associated with reduced adherence and was also associated with younger age, marked baseline immunosuppression, and male sex in the primary model. These findings support the need for targeted interventions focused on adherence support, early diagnosis, and differentiated long-term follow-up of patients.

## 1. Introduction

HIV infection remains one of the leading global public health challenges despite the substantial expansion of testing coverage and ART. According to the Joint United Nations Programme on HIV/AIDS (UNAIDS), by the end of 2024, there were 40.8 million PLHIV worldwide, 31.6 million were receiving ART, 87% knew their HIV status, and 73% of all PLHIV had suppressed viral load [1]. These indicators reflect substantial progress, but at the same time, they point to a persistent gap between expanded access to treatment and the achievement of stable virological control at the population level [1,2].

Virological suppression is considered a key clinical and public health outcome of ART. Achieving and maintaining a low or undetectable VL is associated with a better individual prognosis, a lower risk of disease progression, and prevention of HIV transmission. Therefore, VL monitoring and adherence support remain central components of the contemporary model of care for PLHIV [3].

Virological non-suppression is multifactorial. Systematic reviews and analytical studies have shown that its most consistent predictors include inadequate adherence, stigma, the need to conceal treatment, psychoactive substance use, structural barriers to care, and unfavorable psychosocial conditions [4,5,6,7]. In addition, the risk of VNS is higher among patients with a lower baseline CD4 cell count, which underscores the importance of delayed treatment initiation and accumulated immunosuppression [8].

This issue is of particular importance in Eastern Europe and Central Asia, a region that continues to lag behind the targets of the global HIV care cascade. According to UNAIDS, in 2024, the region was home to 2.1 million PLHIV, 130,000 new infections were registered, and 48,000 acquired immunodeficiency syndrome-related deaths occurred. At the same time, only 51% of all PLHIV were receiving treatment, and only 43% of all PLHIV had suppressed VL [9]. Publications focused on this region point to a combination of late diagnosis, uneven coverage of key populations, organizational barriers, and incomplete retention in care [2,10].

Kazakhstan has demonstrated notable progress in recent years toward achieving the 95-95-95 targets, but the clinical and epidemiological situation remains heterogeneous. According to nationally presented UNAIDS data for 2024, approximately 43,000 PLHIV were living in the country, with an uncertainty range from 35,000 to 46,000. Of these, 82% knew their status, 90% of those who knew their status were receiving ART, and 94% of those receiving treatment had achieved VS [11]. At the same time, the epidemic remains concentrated in key populations, especially among people who inject drugs, and molecular epidemiological studies conducted in Kazakhstan have demonstrated the presence of clinically significant drug resistance mutations that may affect treatment effectiveness [12,13]. This makes it necessary to analyze factors associated with VNS under conditions of real-world clinical practice.

The objective of this study was to assess the prevalence of VS and to identify factors associated with its absence among PLHIV who are receiving ART in Kazakhstan.

## 2. Materials and Methods

### 2.1. Study Design and Analytical Sample

A retrospective cross-sectional analytical study was conducted using secondary analysis of a national registry database of PLHIV receiving ART in the Republic of Kazakhstan as of 30 September 2025. The unit of observation was the individual patient. The initial extraction included 33,614 records.

**Figure 1:**
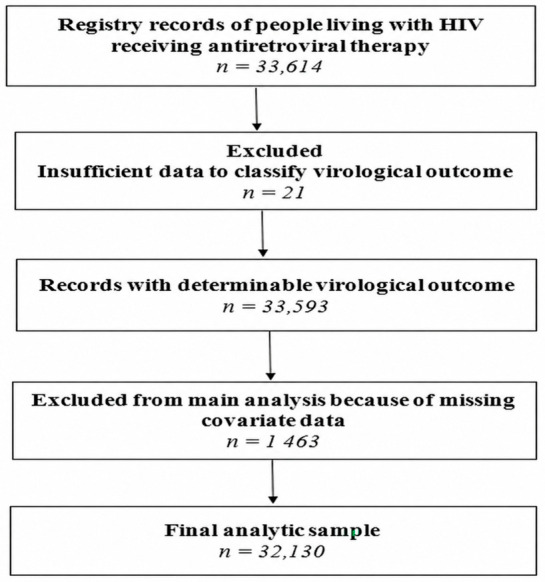
Flowchart of participant inclusion in the analytical sample.

The main analysis included patients registered as receiving ART and having sufficient information to determine the virological outcome on the basis of the last available VL result. The analytical sample used to identify factors associated with VNS was formed using a complete-case approach. The final analytical sample for multivariable analysis consisted of 32,130 patients (Figure 1).

Of 33,614 registry records, 33,593 had sufficient information to determine VS status, and 32,130 were included in the final multivariable analysis after exclusion of records with missing covariate data.

### 2.2. Data Source and Variables

The analysis used de-identified registry data, including sociodemographic characteristics, information on the presumed route of HIV transmission, clinical and laboratory indicators, and parameters of ART. Variables with clinical and epidemiological relevance and acceptable completeness were included in the analytical database.

Sociodemographic characteristics included age and sex. Age was analyzed in the following categories: younger than 25 years, 25 to 34 years, 35 to 44 years, 45 to 54 years, and 55 years or older.

Epidemiological characteristics included the presumed route of HIV transmission. To improve statistical stability, the original categories were consolidated into the following analytical groups: heterosexual transmission, homosexual transmission, injection-related transmission, vertical transmission, and other or unspecified routes.

Clinical characteristics included the first CD4 value available in the registry, hereafter referred to as the first CD4 count. For analysis, this indicator was categorized as follows: <200, 200–349, 350–499, and ≥500 cells/μL.

Treatment-related characteristics included the last recorded percentage category of adherence to ART and the aggregated category of ART regimen. Adherence was analyzed in three categories: <85%, 85–94%, and ≥95%. Adherence was treated as a routine clinical indicator recorded at the time of the last available observation. Observations with missing adherence values were not included in the main analytical dataset.

The ART regimen category reflected the class of the key drug in the current treatment regimen. For analysis, three aggregated groups were defined: DTG-containing regimens, regimens containing NNRTIs, and regimens containing PIs. Regimens included in these groups included, for example, tenofovir disoproxil fumarate with lamivudine and dolutegravir, dolutegravir with abacavir and lamivudine, zidovudine with lamivudine plus dolutegravir, and tenofovir disoproxil fumarate with emtricitabine plus dolutegravir for DTG-containing regimens; emtricitabine with rilpivirine and tenofovir alafenamide, tenofovir disoproxil fumarate with emtricitabine plus etravirine, abacavir with lamivudine plus etravirine, zidovudine with lamivudine plus etravirine, as well as regimens based on efavirenz or nevirapine for regimens containing NNRTIs; and tenofovir disoproxil fumarate with emtricitabine plus darunavir boosted with cobicistat, zidovudine with lamivudine plus darunavir boosted with cobicistat, abacavir with lamivudine plus darunavir boosted with cobicistat, and lamivudine plus darunavir boosted with cobicistat for regimens containing PIs. Mixed and non-standard regimens were classified according to a pre-formed aggregated treatment regimen category.

### 2.3. Study Outcome

The primary outcome was VNS. VS status was determined on the basis of the last recorded VL result using two registry fields: a categorical field reflecting the type of the last result and the numerical value of the last VL.

Patients were classified as having VS if they had an undetectable VL or if the last VL value was <200 copies/mL. VNS was defined as the last VL value of ≥200 copies/mL. The cut-off of 200 copies/mL was selected a priori to identify clinically meaningful virological non-suppression in routine national registry data, rather than strict assay-level detectability or the global programmatic 95-95-95 indicator [14]. This threshold is consistent with clinical guidance that regards failure to achieve or maintain HIV RNA < 200 copies/mL as virologically meaningful, while WHO/UNAIDS programmatic monitoring commonly uses <1000 copies/mL for population-level viral suppression indicators [3,15]. Therefore, the ≥200 copies/mL cut-off was considered appropriate for this registry-based analysis, as it balances clinical relevance with robustness of classification and avoids overclassifying very low-level viraemia as non-suppression.

### 2.4. Data Preparation

Before analysis, the database was cleaned. Duplicate records, plausible ranges of quantitative indicators, correctness of date formats, and logical consistency of related variables were checked. When a discrepancy existed between the categorical type of VL and the numerical value, the numerical value was used to classify the outcome if it allowed an unambiguous determination of VS status. Rare transmission categories were merged into the aggregated group “other or unspecified”. Derived categorical variables were created for age, route of transmission, first CD4 count, adherence, and treatment regimen category.

### 2.5. Statistical Analysis

Statistical analysis was performed using IBM SPSS Statistics, version 24.0. Descriptive results are presented as absolute numbers and proportions for categorical variables. In the main analytical sample, the overall proportions of patients with and without virological suppression were calculated.

At the first stage, categorical variables were compared according to virological suppression status using the Pearson chi-square test; Fisher’s exact test was applied when necessary. In addition, bivariate logistic regression analyses were performed to estimate crude associations between each predictor and virological non-suppression. The results of these analyses are presented as crude odds ratios (cORs) with 95% confidence intervals (CIs).

At the second stage, a multivariable logistic regression model was constructed in which the dependent variable was virological non-suppression. The final model included age, sex, route of HIV transmission, first CD4 count, the last recorded percentage category of adherence to ART, and the aggregated category of ART regimen. Model results are presented as adjusted odds ratios (aORs) with 95% CIs. Associations with a two-sided *p* value < 0.05 were considered statistically significant.

To evaluate possible overlap and collinearity between sex and route of HIV transmission, cross-tabulations were examined, and collinearity diagnostics were performed using variance inflation factors. In addition, a sensitivity analysis was conducted using broader transmission categories. Heterosexual and homosexual transmission were combined into a single sexual transmission category and compared with injection-related transmission and vertical/other or unspecified transmission. This model included the same covariates as the main multivariable model.

To assess the robustness of the strong association between adherence and virological non-suppression, an additional sensitivity analysis was performed using a collapsed binary adherence variable. Patients with adherence of 85–94% and <85% were combined into a single < 95% adherence category and compared with patients with adherence ≥ 95%. This analysis was conducted to evaluate whether the observed association was sensitive to the original three-category parameterization of adherence and to assess robustness in the presence of imbalance across adherence categories. The sensitivity model included the same covariates as the main multivariable model, with the collapsed adherence variable replacing the original three-category adherence variable.

To explore the unexpected association between ART regimen category and virological non-suppression, additional exploratory analyses were performed by comparing adherence, first CD4 count, treatment line, duration since ART initiation, and recorded drug resistance across regimen categories. A sensitivity analysis restricted to patients with documented adherence ≥ 95% was also conducted. In addition, an extended model further adjusted for duration since ART initiation and a treatment line was fitted to assess whether these factors attenuated the association between ART regimen category and virological non-suppression.

Missing data were assessed separately for the outcome and for all covariates included in the analysis. The main descriptive, bivariate, and multivariable analyses were conducted using a complete-case approach and included only patients with complete data on the virological outcome and on all covariates included in the final model.

The original registry database contained 33,614 records. In 21 patients, data sufficient to classify the virological outcome were missing. Among patients with a determinable virological status, 1463 records were excluded from the main analytical dataset because of missing or unusable covariate values for the final model. Thus, the final analytical sample for descriptive, bivariate, and multivariable analyses consisted of 32,130 patients. Missing values were not imputed.

## 3. Results

### 3.1. Analytical Sample and Prevalence of Virological Non-Suppression

The initial registry extraction included 33,614 records. After exclusion of observations with missing data on the outcome and on covariates included in the main analysis, the final analytical sample comprised 32,130 patients. Descriptive, bivariate, and multivariable analyses were performed on this sample. VS was achieved in 29,454 (91.7%) patients, whereas VNS was recorded in 2676 (8.3%) patients.

### 3.2. Bivariate Distribution of Virological Non-Suppression

In the bivariate analysis, VNS was significantly associated with age, sex, route of HIV transmission, first CD4 count, level of adherence to ART, and type of treatment regimen.

The proportion of patients without VS decreased progressively with increasing age, from 10.1 percent among patients younger than 25 years to 6.3 percent among those aged 55 years or older (*p* < 0.001). VNS was more frequent among men than among women, 8.7 percent versus 7.7 percent (*p* = 0.001).

Statistically significant differences were also observed according to the presumed route of HIV transmission. The lowest proportion of VNS was observed among men who have sex with men, at 5.5%, whereas the corresponding proportions were 8.5% among patients with heterosexual transmission and 8.6% among people who inject drugs. The proportion of VNS was 7.7% among patients with vertical transmission and 7.6% among those with other or unspecified transmission routes.

Baseline immunological status was also associated with the virological outcome. Among patients with a first CD4 count < 200 cells/μL, the proportion of non-suppression was 9.4%, whereas in the 200–349, 350–499, and ≥500 cells/μL groups it was 8.3%, 8.2%, and 7.8%, respectively (*p* = 0.007).

The most pronounced differences were observed by level of adherence to therapy (*p* < 0.001). Among patients with adherence ≥ 95%, the absence of virological suppression was observed in 3.7% of cases, whereas it was 52.6% in those with adherence of 85–94% and 70.1% in those with adherence < 85%.

The type of ART regimen was also significantly associated with the virological outcome (*p* < 0.001). The proportion of VNS was 8.5% among patients receiving DTG-containing regimens, 4.4% among patients receiving regimens containing NNRTIs, and 9.4% among patients receiving regimens containing PIs. The summary bivariate results are presented in Table 1.

### 3.3. Crude and Adjusted Factors Associated with Virological Non-Suppression

Bivariate logistic regression analyses were performed to estimate crude associations between each predictor and virological non-suppression before multivariable adjustment. The crude and adjusted estimates are presented together in Table 2. A total of 32,130 patients were included in the multivariable logistic regression model. After mutual adjustment for sex, age, route of human immunodeficiency virus transmission, first CD4 count, adherence to antiretroviral therapy, and type of treatment regimen, the absence of virological suppression remained significantly associated with several factors (Table 2). Men had higher adjusted odds of virological non-suppression than women (adjusted odds ratio 1.14; 95% confidence interval 1.02–1.26; *p* = 0.017). Compared with patients younger than 25 years, the adjusted odds of virological non-suppression were lower in all older age groups: 25–34 years (adjusted odds ratio 0.61; 95% confidence interval 0.46–0.82; *p* < 0.001), 35–44 years (adjusted odds ratio 0.56; 95% confidence interval 0.42–0.74; *p* < 0.001), 45–54 years (adjusted odds ratio 0.46; 95% confidence interval 0.35–0.61; *p* < 0.001), and ≥55 years (adjusted odds ratio 0.42; 95% confidence interval 0.31–0.57; *p* < 0.001).

Among clinical factors, only the first CD4 count < 200 cells/μL remained independently associated with higher adjusted odds of virological non-suppression compared with a CD4 count ≥ 500 cells/μL (adjusted odds ratio 1.25; 95% confidence interval 1.09–1.44; *p* = 0.002). The strongest association was observed for adherence to therapy: compared with patients with adherence ≥ 95%, the adjusted odds of virological non-suppression were significantly higher in those with adherence of 85–94% (adjusted odds ratio 28.66; 95% confidence interval 25.85–31.77; *p* < 0.001) and <85% (adjusted odds ratio 61.05; 95% confidence interval 50.50–73.81; *p* < 0.001). Among treatment regimens, NNRTI-containing regimens were associated with lower adjusted odds of virological non-suppression compared with DTG-containing regimens (adjusted odds ratio 0.68; 95% confidence interval 0.52–0.88; *p* = 0.004), whereas no statistically significant differences were identified for PI-containing regimens (adjusted odds ratio 1.01; 95% confidence interval 0.83–1.22; *p* = 0.948). Compared with heterosexual transmission, lower adjusted odds of virological non-suppression were observed among patients with homosexual transmission (adjusted odds ratio 0.65; 95% confidence interval 0.52–0.82; *p* < 0.001), vertical transmission (adjusted odds ratio 0.41; 95% confidence interval 0.25–0.68; *p* < 0.001), and other or unspecified transmission routes (adjusted odds ratio 0.60; 95% confidence interval 0.39–0.90; *p* = 0.015), whereas no differences were found for injection-related transmission compared with heterosexual transmission (adjusted odds ratio 0.91; 95% confidence interval 0.81–1.02; *p* = 0.121). Intermediate categories of first CD4 count (200–349 and 350–499 cells/μL) also did not remain significantly associated with the outcome after adjustment for other factors. The strongest independent association was observed for reduced adherence to therapy, particularly in the group with adherence < 85%.

The multivariable model simultaneously included sex, age group, route of HIV transmission, first CD4 count, adherence to ART, and type of treatment regimen.

Because the adherence categories were highly imbalanced and the adjusted odds ratios for reduced adherence were large, an additional sensitivity analysis was conducted using a collapsed binary adherence variable. Patients with adherence of 85–94% and <85% were combined into a single < 95% adherence category and compared with those with adherence ≥ 95%. In this analysis, virological non-suppression was observed in 56.2% of patients with adherence < 95% compared with 3.7% of those with adherence ≥ 95%. The association remained strong in both crude and adjusted models. In the multivariable model, adherence < 95% was associated with substantially higher odds of virological non-suppression compared with adherence ≥ 95% (aOR 33.09; 95% CI 30.05–36.45; *p* < 0.001). This finding supports the robustness of the main adherence-related results. The detailed results of this sensitivity analysis are presented in Supplementary Table S1.

Because sex and route of HIV transmission may partly capture overlapping epidemiological structures, particularly for homosexual transmission, additional checks were performed. Cross-tabulation showed that the homosexual transmission category was almost entirely composed of men, whereas the heterosexual, vertical, and other or unspecified categories included both men and women. Collinearity diagnostics did not indicate problematic multicollinearity between sex and transmission route. In a sensitivity analysis using broader transmission categories, injection-related transmission did not differ significantly from sexual transmission in the adjusted model (aOR 0.95; 95% CI 0.84–1.06; *p* = 0.351), whereas vertical/other or unspecified transmission was associated with lower odds of virological non-suppression (aOR 0.56; 95% CI 0.40–0.80; *p* = 0.001). In this model, the association between male sex and virological non-suppression was attenuated and was no longer statistically significant (aOR 1.08; 95% CI 0.98–1.20; *p* = 0.127). These findings indicate that sex- and transmission-related associations should be interpreted cautiously.

### 3.4. Exploratory and Sensitivity Analyses by ART Regimen Category

To further examine the unexpected association between ART regimen category and virological non-suppression, additional exploratory analyses were conducted across the three regimen groups. Patients receiving NNRTI-containing regimens had a more favorable clinical and treatment-related profile than those receiving DTG-containing regimens. The proportion of patients with documented adherence ≥ 95% was higher in the NNRTI group than in the DTG group, whereas the proportions with adherence of 85–94% and <85% were lower. The NNRTI group also had a higher proportion of patients with a first CD4 count ≥ 500 cells/μL and a longer median duration since ART initiation. By contrast, PI-containing regimens were more frequently observed among patients with lower adherence, first CD4 count < 200 cells/μL, second-line treatment, and recorded drug resistance. These exploratory comparisons are presented in Supplementary Table S2.

In a sensitivity analysis restricted to patients with documented adherence ≥ 95%, the absolute prevalence of virological non-suppression was low across all regimen categories. Virological non-suppression was observed in 3.8% of patients receiving DTG-containing regimens, 2.5% of those receiving NNRTI-containing regimens, and 3.1% of those receiving PI-containing regimens. In this subgroup, NNRTI-containing regimens remained associated with lower odds of virological non-suppression compared with DTG-containing regimens, although the absolute difference between groups was small. In an additional model adjusted for duration since ART initiation and treatment line, the association between NNRTI-containing regimens and lower odds of virological non-suppression was attenuated and was no longer statistically significant (aOR 0.83; 95% CI 0.63–1.09; *p* = 0.184). PI-containing regimens were also not significantly associated with virological non-suppression in this extended model (aOR 1.14; 95% CI 0.94–1.38; *p* = 0.195). The full model outputs for the broader transmission-route sensitivity analysis and the ART-regimen sensitivity analyses are presented in Supplementary Tables S3–S5.

## 4. Discussion

This study provides national real-world clinical data on virological non-suppression among people living with human immunodeficiency virus who are receiving antiretroviral therapy in Kazakhstan. Overall, virological suppression was achieved in 91.7% of patients included in the final analytical sample. This proportion indicates a high level of virological control under routine care conditions and is generally consistent with recent data from Kazakhstan, as well as with broader estimates suggesting continued, although uneven, progress among countries in Central and Eastern Europe toward achieving HIV treatment targets. At the same time, a clinically meaningful subgroup of patients remains without virological suppression. In this sense, our findings add to the existing picture by showing that the residual burden of non-suppression is not randomly distributed, but rather concentrated in specific demographic, clinical, and treatment-related subgroups, underscoring the need for more targeted programmatic interventions [16,17].

The most important finding of the present study is the exceptionally strong association between reduced adherence and virological non-suppression. After mutual adjustment for other factors, reduced adherence remained the strongest factor associated with virological non-suppression, with markedly higher adjusted odds among patients with adherence of 85–94% (aOR 28.66; 95% CI 25.85–31.77) and <85% (aOR 61.05; 95% CI 50.50–73.81) compared with those with adherence ≥ 95%. This result is fully consistent with the broader literature, in which adherence remains one of the most stable and modifiable determinants of virological control. The literature shows that treatment interruptions, stigma, the need to conceal medication use, psychoactive substance use, structural barriers to care, and other psychosocial constraints reduce adherence and thereby increase the likelihood of virological failure [18,19]. Consequently, our findings further indicate that virological non-suppression in routine practice is closely linked to behavioral and programmatic aspects of patient management, rather than being solely a pharmacological issue.

The age gradient identified in our data also deserves attention. In the adjusted analysis, the adjusted odds of virological non-suppression were lower in all age groups older than 25 years than among patients younger than 25 years, with the lowest adjusted odds observed among patients aged ≥55 years [20,21]. This is consistent with previous studies showing that younger age is associated with delayed treatment initiation, lower adherence, and a higher risk of virological rebound or failure to achieve suppression. Because adherence was included in the multivariable model, the age-related association should not be attributed to adherence alone. Younger age may also capture broader psychosocial, behavioral, and structural factors that influence engagement in HIV care, including stigma, lower perceived risk, unstable daily routines, mobility, work or study-related barriers, and limited experience with long-term treatment [21,22]. Thus, younger patients may require youth-sensitive retention strategies, flexible appointment systems, targeted adherence support, and timely follow-up after missed visits.

Male sex also remained independently associated with higher adjusted odds of non-suppression after adjustment. Overall, this finding is consistent with studies of routine HIV care programs in low- and middle-income countries, where men often demonstrate less favorable outcomes at various stages of the care cascade, including later presentation to care, less stable retention in follow-up, and a lower likelihood of sustained virological suppression [23,24]. From a practical perspective, the sex differences identified in our study are unlikely to reflect only a biological mechanism; rather, they probably capture broader gender differences in health care seeking, continuity of follow-up, and engagement in treatment [25]. For Kazakhstan, this finding indicates the need to strengthen viral load monitoring and adherence support, especially among working-age men, who may be retained less consistently in a system of structured long-term follow-up.

The association between male sex and virological non-suppression should be interpreted as a marker of possible programmatic vulnerability rather than as a biological effect. In the sociocultural context of Kazakhstan, this may reflect delayed testing, work-related barriers to clinic attendance, stigma, fear of disclosure, and less consistent long-term engagement in HIV care among men. Therefore, men of working age may require more flexible and patient-centered retention strategies, including appointment scheduling adapted to work constraints, proactive follow-up after missed visits, and targeted adherence support.

Among clinical factors, only marked baseline immunosuppression—defined in our study as a first CD4 count below 200 cells/μL—remained independently associated with non-suppression in the final model. This result is important because it shows that the influence of baseline immunological status is not merely descriptive but continues to shape virological outcomes even after treatment initiation [26]. This pattern is consistent with previous studies showing that a lower baseline CD4 count is associated with a greater propensity toward virological failure, probably reflecting the consequences of late diagnosis, delayed treatment initiation, more advanced disease stage, and an overall more complex clinical course [8,27]. In this regard, our findings support strengthening early HIV diagnosis and timely treatment initiation as key programmatic priorities for reducing the risk of subsequent virological non-suppression.

The associations identified for transmission categories and types of antiretroviral therapy regimen require particularly cautious interpretation. After adjustment, patients with homosexual and vertical routes of transmission, as well as those classified in the other or unspecified transmission category, had lower adjusted odds of non-suppression than patients with heterosexual transmission, whereas no statistically significant differences were identified for injection-related transmission. These findings should not be interpreted as indicating intrinsic biological differences in risk between transmission categories. More likely, they reflect differences in engagement in care, timing of diagnosis, nature of treatment support, and social composition of the subgroups represented in the registry [10]. This interpretation is consistent with broader evidence from Europe and Central Asia showing that the HIV care cascade varies substantially across key populations and social contexts [17].

The absence of a statistically significant difference for injection-related transmission should also be interpreted cautiously. This finding does not reduce the epidemiological importance of people who inject drugs in Kazakhstan or the wider Eastern Europe and Central Asia region. It may reflect earlier identification through targeted testing, harm-reduction services, or established linkage-to-care pathways among patients who had already entered ART care. However, because the registry did not include individual-level information on harm-reduction program participation, timing of diagnosis, or linkage-to-care pathways, this interpretation remains hypothesis-generating.

Similarly, the lower adjusted odds of non-suppression among patients receiving NNRTI-containing regimens compared with those receiving DTG-containing regimens should not be interpreted as evidence of greater virological efficacy of NNRTI-based therapy. Contemporary evidence generally supports high levels of suppression with dolutegravir-containing treatment [28,29], and the apparent advantage of NNRTI-containing regimens in our data most likely reflects treatment selection patterns, selection of patients who remained stable on long-term regimens, or other forms of confounding by indication. This point is particularly important for Kazakhstan, where molecular epidemiological studies have already demonstrated the presence of clinically significant drug resistance mutations that may complicate the interpretation of routine data [12,13].

From a programmatic perspective, the present findings suggest that the remaining burden of virological non-suppression in Kazakhstan should be addressed through differentiated rather than uniform follow-up strategies. Patients with documented suboptimal adherence require priority attention, including structured adherence counselling, timely follow-up after missed visits, and closer viral load monitoring. Younger patients and men may also benefit from more flexible and patient-centered retention strategies, as these groups may face specific barriers to sustained engagement in long-term care. In addition, individuals entering care with marked immunosuppression should be considered a clinically vulnerable group requiring early treatment initiation, careful follow-up, and timely assessment of treatment response. Given the available evidence on drug resistance in Kazakhstan, interpretation of viral load monitoring should also take treatment history into account and, where available, the results of resistance testing. These findings indicate that further progress toward national viral suppression targets will depend not only on ART availability but also on sustained retention in care, systematic adherence support, early diagnosis, and active management of patients at higher risk of virological non-suppression.

The present study has several strengths. It is based on a large national registry and reflects routine clinical practice rather than the more selective conditions of a clinical trial or a single-center cohort. The size of the analytical sample was sufficient to provide stable estimates across several clinically meaningful subgroups. In addition, the study combined demographic, clinical, and treatment-related factors within a single analytical model, making it possible to determine which associations remained after mutual adjustment.

Several limitations should be considered. First, the study had a cross-sectional design and was based on secondary registry data; therefore, the identified associations cannot be interpreted as causal. Second, the analysis depended on the completeness and accuracy of routine registry data. Although duration since ART initiation and treatment line were available for exploratory analyses, detailed longitudinal information on prior ART regimens, reasons for switching, previous virological failure, treatment interruptions, psychosocial factors, mental health, psychoactive substance use, and complete resistance testing results were not available for the primary analysis. Third, the complete-case approach may have introduced selection bias if patients with missing data differed systematically from those included in the final sample. Fourth, adherence was based on a routine registry record and reflected the last available observation; therefore, it should be regarded as a clinical and programmatic indicator rather than a direct causal measure. Fifth, comparisons between aggregated ART regimen categories should be interpreted with caution, because regimen assignment was not random and may reflect prior treatment history, regimen switching, tolerability, adherence challenges, previous treatment failure, drug resistance, and overall clinical complexity. Thus, residual confounding by indication and stable survivor bias cannot be excluded. Sixth, sex and route of HIV transmission may partly overlap, particularly because homosexual transmission was predominantly recorded among men. Although VIF diagnostics did not indicate problematic statistical multicollinearity, this does not fully exclude conceptual overlap between sex and route of HIV transmission. Sensitivity analyses with broader transmission categories attenuated the sex-related association; therefore, sex- and transmission-related findings should be interpreted as subgroup markers rather than causal effects. Seventh, the estimated prevalence of virological non-suppression depends on the viral load threshold used. A stricter threshold, such as ≥50 copies/mL, would likely produce a higher estimate, whereas the WHO/UNAIDS programmatic threshold for non-suppression, such as >1000 copies/mL, would likely produce a lower estimate. Therefore, our findings should be interpreted as clinically meaningful virological non-suppression defined by the ≥200 copies/mL threshold, rather than strict assay-level detectability or programmatic non-suppression. Despite these limitations, the large national registry provided practically important estimates of factors associated with virological non-suppression in real-world HIV care in Kazakhstan.

## 5. Conclusions

Taken together, our findings show that, despite the high overall level of virological suppression among PLHIV who are receiving antiretroviral therapy in Kazakhstan, virological non-suppression remains concentrated in clearly identifiable subgroups, primarily among patients with reduced adherence, younger age, male sex, and marked baseline immunosuppression. These data provide a practical basis for more targeted HIV program strategies focused on adherence support, earlier diagnosis, and differentiated long-term follow-up of patients.

## Figures and Tables

**Table 1:** Sample characteristics and bivariate associations with virological non-suppression (*n* = 32,130).

Variable	Category	Virological Suppression, *n* (%)	Virological Non-Suppression, *n* (%)	Total, *n*	*p* Value
Sex	Women	11,723 (92.3)	980 (7.7)	12,703	0.001
	Men	17,731 (91.3)	1696 (8.7)	19,427	
Age group	<25 years	1214 (89.9)	136 (10.1)	1350	<0.001
	25–34 years	4760 (90.7)	488 (9.3)	5248	
	35–44 years	11,301 (91.1)	1105 (8.9)	12,406	
	45–54 years	8353 (92.4)	690 (7.6)	9043	
	≥55 years	3826 (93.7)	257 (6.3)	4083	
Route of transmission	Heterosexual	18,938 (91.5)	1770 (8.5)	20,708	<0.001
	Injection-related transmission	7540 (91.4)	710 (8.6)	8250	
	Homosexual	2069 (94.5)	121 (5.5)	2190	
	Other or unspecified	486 (92.4)	40 (7.6)	526	
	Vertical	421 (92.3)	35 (7.7)	456	
First CD4 count, cells/μL	<200	5325 (90.6)	552 (9.4)	5877	0.007
	200–349	7429 (91.7)	672 (8.3)	8101	
	350–499	7174 (91.8)	642 (8.2)	7816	
	≥500	9526 (92.2)	810 (7.8)	10,336	
Adherence to ART	<85%	172 (29.9)	404 (70.1)	576	<0.001
	85–94%	1077 (47.4)	1196 (52.6)	2273	
	≥95%	28,205 (96.3)	1076 (3.7)	29,281	
ART regimen	DTG-containing regimens	26,093 (91.5)	2419 (8.5)	28,512	<0.001
	Regimens containing NNRTIs	1580 (95.6)	73 (4.4)	1653	
	Regimens containing PIs	1781 (90.6)	184 (9.4)	1965	

**Table 2:** Crude and adjusted logistic regression of factors associated with virological non-suppression (*n* = 32,130).

Variable	Category	cOR (95% CI)	*p* Value	aOR (95% CI)	*p* Value
Sex	Women	Ref	-	Ref	-
	Men	1.14 (1.05–1.24)	0.001	1.14 (1.02–1.26)	0.017
Age group	<25 years	Ref	-	Ref	-
	25–34 years	0.92 (0.75–1.12)	0.385	0.61 (0.46–0.82)	<0.001
	35–44 years	0.87 (0.72–1.05)	0.155	0.56 (0.42–0.74)	<0.001
	45–54 years	0.74 (0.61–0.89)	0.002	0.46 (0.35–0.61)	<0.001
	≥55 years	0.60 (0.48–0.75)	<0.001	0.42 (0.31–0.57)	<0.001
Route of transmission	Heterosexual	Ref	-	Ref	-
	Injection-related transmission	1.01 (0.92–1.10)	0.872	0.91 (0.81–1.02)	0.121
	Homosexual	0.63 (0.52–0.76)	<0.001	0.65 (0.52–0.82)	<0.001
	Vertical	0.89 (0.63–1.26)	0.510	0.41 (0.25–0.68)	<0.001
	Other or unspecified	0.88 (0.64–1.22)	0.445	0.60 (0.39–0.90)	0.015
First CD4 count, cells/μL	≥500	Ref	-	Ref	-
	350–499	1.05 (0.94–1.17)	0.354	1.05 (0.92–1.19)	0.499
	200–349	1.06 (0.96–1.18)	0.256	1.05 (0.92–1.20)	0.443
	<200	1.22 (1.09–1.37)	0.001	1.25 (1.09–1.44)	0.002
Adherence to ART	≥95%	Ref	-	Ref	-
	85–94%	29.11 (26.28–32.25)	<0.001	28.66 (25.85–31.77)	<0.001
	<85%	61.57 (50.99–74.35)	<0.001	61.05 (50.50–73.81)	<0.001
ART regimen	DTG-containing regimens	Ref	-	Ref	-
	Regimens containing NNRTIs	0.50 (0.39–0.63)	<0.001	0.68 (0.52–0.88)	0.004
	Regimens containing PIs	1.11 (0.95–1.30)	0.177	1.01 (0.83–1.22)	0.948

## Data Availability

All available data are presented within the manuscript.

## Supplementary Materials

The following supporting information can be downloaded at: https://www.mdpi.com/article/10.3390/tropicalmed11060156/s1, Table S1: Sensitivity analysis using collapsed adherence categories. Table S2: Clinical and treatment-related characteristics by ART regimen category. Table S3: Full sensitivity model using broader transmission route categories. Table S4: Full ART regimen sensitivity model restricted to patients with adherence ≥ 95%. Table S5: Full extended ART regimen model additionally adjusted for ART duration and treatment line.

## Abbreviations

The following abbreviations are used in this manuscript:

aOR	Adjusted Odds Ratio
ART	Antiretroviral Therapy
CD4	Cluster of Differentiation 4
CI	Confidence Interval
DTG	Dolutegravir
HIV	Human Immunodeficiency Virus
NNRTI	Non-Nucleoside Reverse Transcriptase Inhibitor
PI	Protease Inhibitor
PLHIV	People Living with HIV
SPSS	Statistical Package for the Social Sciences
UNAIDS	Joint United Nations Programme on HIV/AIDS
VL	Viral Load
VNS	Virological Non-Suppression
VS	Virological Suppression
