# Supplementary material for: Factors Associated with Virological Non-Suppression Among People Living with HIV Receiving Antiretroviral Therapy in Kazakhstan: A National Registry-Based Study
Source: Trop Med Infect Dis. 2026 Jun 9;11(6):156. doi: 10.3390/tropicalmed11060156 (PMC13307818; doi:10.3390/tropicalmed11060156)
Supplement: Supplementary file 1 [file tropicalmed-11-00156-s001.zip › tropicalmed-4316411-supplementary.pdf]

Supplementary Table S1. Sensitivity analysis using collapsed adherence categories.

**Adherence category**	**VS, n (%)**	**VNS, n (%)**	**cOR (95% CI)**	**p value**	**aOR (95% CI)**	**p value**
≥95%	28 205 (96.3)	1 076 (3.7)	Ref	-	Ref	-
<95%	1 249 (43.8)	1 600 (56.2)	33.58 (30.51-36.96)	<0.001	33.09 (30.05-36.45)	<0.001

Supplementary Table S2. Clinical and treatment-related characteristics by ART regimen category.

**Characteristic**	**DTG-containing regimens, n = 28 512**	**NNRTI-containing regimens, n = 1 653**	**PI-containing regimens, n = 1 965**
Adherence ≥95%, n (%)	25 952 (91.0)	1 575 (95.3)	1 754 (89.3)
Adherence 85-94%, n (%)	2 037 (7.1)	68 (4.1)	168 (8.5)
Adherence <85%, n (%)	523 (1.8)	10 (0.6)	43 (2.2)
First CD4 <200 cells/μL, n (%)	5 192 (18.2)	269 (16.3)	416 (21.2)
First CD4 ≥500 cells/μL, n (%)	9 155 (32.1)	610 (36.9)	571 (29.1)
Second-line treatment, n (%)	954 (3.3)	72 (4.4)	149 (7.6)
Duration since ART initiation, median (IQR), years	5.09 (2.56-7.84)	7.35 (4.86-9.95)	6.59 (3.53-9.17)
Recorded drug resistance, n (%)	1 446 (5.1)	73 (4.4)	237 (12.1)

Values are presented as n (%) unless otherwise indicated. Duration since ART initiation is presented as median (interquartile range).

Supplementary Table S3. Full sensitivity model using broader transmission route categories

Variable	Category	Total n	VNS n	aOR (95% CI)	p value
Sex	Women	12 703	980	Ref	-
	Men	19 427	1 696	1.08 (0.98-1.20)	0.127
Age group	<25 years	1 350	136	Ref	-
	25-34 years	5 248	488	0.68 (0.51-0.90)	0.006
	35-44 years	12 406	1 105	0.65 (0.49-0.85)	0.001
	45-54 years	9 043	690	0.53 (0.41-0.70)	<0.001
	≥55 years	4 083	257	0.49 (0.36-0.66)	<0.001
Broader route of transmission	Sexual transmission	22 898	1 891	Ref	-
	Injection-related transmission	8 250	710	0.95 (0.84-1.06)	0.351
	Vertical/other or unspecified	982	75	0.56 (0.40-0.80)	0.001
First CD4 count, cells/µL	≥500	10 336	810	Ref	-
	350-499	7 816	642	1.05 (0.92-1.19)	0.490
	200-349	8 101	672	1.05 (0.92-1.19)	0.494
	<200	5 877	552	1.25 (1.09-1.44)	0.001
Adherence to ART	≥95%	29 281	1 076	Ref	-
	85-94%	2 273	1 196	28.94 (26.11-32.09)	<0.001
	<85%	576	404	61.79 (51.11-74.69)	<0.001
ART regimen	DTG-containing regimens	28 512	2 419	Ref	-
	Regimens containing NNRTIs	1 653	73	0.68 (0.52-0.88)	0.004
	Regimens containing PIs	1 965	184	1.01 (0.83-1.22)	0.951

Sexual transmission includes heterosexual and homosexual transmission. The model included sex, age group, broader route of HIV transmission, first CD4 count, adherence to ART, and ART regimen category.

Supplementary Table S4. Full ART regimen sensitivity model restricted to patients with adherence ≥95%
n = 29 281, VNS = 1 076.

**Variable**	**Category**	**Total n**	**VNS n**	**aOR (95% CI)**	**p value**
Sex	Women	11 661	375	Ref	-
	Men	17 620	701	1.33 (1.16-1.53)	<0.001
Age group	<25 years	1 216	70	Ref	-
	25-34 years	4 753	204	0.46 (0.34-0.64)	<0.001
	35-44 years	11 252	416	0.37 (0.28-0.51)	<0.001
	45-54 years	8 256	268	0.32 (0.23-0.44)	<0.001
	≥55 years	3 804	118	0.30 (0.21-0.42)	<0.001
Route of transmission	Heterosexual	18 894	745	Ref	-
	Injection-related transmission	7 424	246	0.83 (0.71-0.97)	0.018
	Homosexual	2 083	61	0.51 (0.38-0.67)	<0.001
	Vertical	407	12	0.32 (0.17-0.61)	<0.001
	Other or unspecified	473	12	0.43 (0.23-0.80)	0.007
First CD4 count, cells/μL	≥500	9 448	319	Ref	-
	350-499	7 134	235	0.96 (0.81-1.14)	0.666
	200-349	7 380	273	1.09 (0.92-1.28)	0.331
	<200	5 319	249	1.43 (1.20-1.71)	<0.001
ART regimen	DTG-containing regimens	25 952	983	Ref	-
	Regimens containing NNRTIs	1 575	39	0.69 (0.50-0.96)	0.026
	Regimens containing PIs	1 754	54	0.83 (0.63-1.10)	0.193

The analysis was restricted to patients with documented adherence ≥95%. The model included sex, age group, route of HIV transmission, first CD4 count, and ART regimen category.

Supplementary Table S5. Full extended ART regimen model additionally adjusted for ART duration and treatment line

n = 32 000, VNS = 2 627.

**Variable**	**Category**	**Total n**	**VNS n**	**aOR (95% CI)**	**p value**
Sex	Women	12 660	963	Ref	-
	Men	19 340	1 664	1.03 (0.92-1.14)	0.628
Age group	<25 years	1 346	136	Ref	-
	25-34 years	5 225	480	0.67 (0.50-0.89)	0.006
	35-44 years	12 354	1 082	0.68 (0.51-0.89)	0.006
	45-54 years	9 011	679	0.59 (0.44-0.78)	<0.001
	≥55 years	4 064	250	0.52 (0.38-0.71)	<0.001
Route of transmission	Heterosexual	20 633	1 741	Ref	-
	Injection-related transmission	8 204	690	0.98 (0.87-1.11)	0.766
	Homosexual	2 184	121	0.66 (0.52-0.83)	<0.001
	Vertical	455	35	0.98 (0.59-1.63)	0.951
	Other or unspecified	524	40	1.05 (0.68-1.60)	0.838
First CD4 count, cells/µL	≥500	10 282	789	Ref	-
	350-499	7 781	625	1.05 (0.92-1.20)	0.468
	200-349	8 075	666	1.07 (0.94-1.22)	0.312
	<200	5 862	547	1.22 (1.06-1.40)	0.006
Adherence to ART	≥95%	29 198	1 065	Ref	-
	85-94%	2 236	1 168	25.47 (22.92-28.31)	<0.001
	<85%	566	394	53.22 (43.81-64.66)	<0.001
ART regimen	DTG-containing regimens	28 403	2 375	Ref	-
	Regimens containing NNRTIs	1 644	71	0.83 (0.63-1.09)	0.184
	Regimens containing PIs	1 953	181	1.14 (0.94-1.38)	0.195
Treatment line	First-line treatment	30 831	2 555	Ref	-
	Second-line treatment	1 169	72	0.87 (0.65-1.17)	0.374
Duration since ART initiation	Per one-year increase	32 000	2 627	0.87 (0.85-0.88)	<0.001

The extended model was based on complete cases for all variables included in the model, including duration since ART initiation and treatment line. For duration since ART initiation, the odds ratio is presented per one-year increase.
